# The Principle Underlying the Scottish Registration Bill

**Published:** 1909-03-13

**Authors:** 


### Nursing Administration.

### THE PRINCIPLE UNDERLYING THE SCOTTISH REGISTRATION BILL.

### CONTROL OF NURSING EDUCATION.

The interest excited in Scotland by the draft Registration Bill lately brought under the notice of the public far exceeds any that has ever been exhibited in England on the various occasions when efforts have been made to a similar end. The real point at issue, however, is not so much the question whether or no nurses shall be registered, whether or no Scotland shall have its own Bill, but whether nursing education in Scotland shall continue in the same hands as at present, or pass under the control of a distant body, imperfectly acquainted with the conditions affecting the Scotch nurses whose training they will regulate. Drawn into the open, this is the crux of the whole business, whether it be looked at from the Edinburgh, London, Dublin, Liverpool, or Cardiff point of view. It is this which accounts for the opposition accorded to the theory of registration in England. It is this which leads Scotch matrons and medical superintendents to promote a Bill applying exclusively to Scotland. It is no mere exhibition of professional caution or professional jealousy. In order to understand the keen feeling evoked, it is necessary to inquire what is the object of registration for nurses? The two main reasons which might render it expedient to institute a register are, first, to reform nursing education, and, secondly, to distinguish trained nurses from nurses who are untrained. Which is the view most consistently kept before the public? Undoubtedly the "cry" upon which registration comes before the public is the second, the hardships of highly trained nurses being confused with ignorant impostors, the dangers to which the public is exposed by confusing the one for the other. So far as we have been able to observe, no one has ever ventured to maintain that nurses trained in reputable hospitals granting three-year certificates are not generally efficient. Doctors do not complain that they cannot get skilled nurses because the training schools are at fault. On the contrary, the doctors are perpetually sinning by employing women who have had no training whatever. The public does not complain that the nurses it employs are not sufficiently instructed in anatomy or chemistry. On the contrary, they evade the obligation of engaging women who have gone through a regular course of instruction whenever they can. There is absolutely no demand in the present day for improving the training of nurses, except among those who have all along laboured in the cause with whole-hearted enthusiasm, the matrons and medical superintendents of the hospitals which are recognised as training schools for nurses. It is to the matrons of the general hospitals in the kingdom that the evolution of the trained nurse as she appears to-day, second to none in the world, is due. In the struggle to educate the probationer, to make her training a reality, and, through her, to raise the whole body of nurses, matrons all over the kingdom have been most generously seconded by the medical and lay authorities. It is thus that nursing education has become a power instead of a curriculum. It is to these women that we must look in the future for many new developments as experience shows them fresh opportunities for improvement. To whom can the regulation of nursing education more properly be entrusted than to those who are at the present moment so ably fulfilling their obligations to their probationers? Now the principle of the Scotch Bill is that the matrons shall retain the control they now possess over the training of nurses. The certificate of the nurse's training school, probably issued after some improved and uniform pattern, is to remain what it has always been, the nurse's passport to employment through life, though endorsed by the public registration body, in order to safeguard the public, and distinguish it unmistakably from certificates of a worthless description. The examination which concludes her term of service in the hospital will continue to be held in her own training school, and will test her abilities through the agency of outside examiners appointed or sanctioned by the registration body. It may be harder in one institution than in another, for why should the general trend of nursing education be whittled down to the lowest level imposed in the interests of those nurses of merely average intelligence who are yet indispensable to the general nursing service? Though possibly with a varying standard, these examinations will never be allowed to fall below the standard of things requisite for the trained nurse to know, and any deficiencies which they expose in the general training of any institution will be rectified by practical inspection and advice.

But the principle of upholding the supremacy of the heads of training schools depends no less on properly constituting the registration body than on respecting the dignity of the training certificate. Under the Scotch scheme it will be possible to secure that this registration body will have the advice of no fewer than seven superintendents of Scotch training schools to regulate the issue of certificates of registration, and the conditions of admission to the register of nurses, to regulate and supervise the course of training and the examinations required to qualify for registration, to appoint inspectors, etc. This means control worth having. It ensures that nursing education in Scotland will be kept up to its present high and eminently practical standard, that modern developments will not be ignored, and that national conditions will be understood. Contrast this measure of representation with the presence on a General Council of Registration sitting in London of one Scotch nurse, who need not even actually be engaged in the business of training nurses, and it will be seen that the Scotch authorities are not contending for empty legal distinctions, but for the most vital principles which lie at the root of their magnificent training schools for nurses.

